# Supplementary material for: Mathematical model of COVID-19 intervention scenarios for São Paulo—Brazil
Source: Nat Commun. 2021 Jan 18;12:418. doi: 10.1038/s41467-020-20687-y (PMC7814036; doi:10.1038/s41467-020-20687-y)
Supplement: Supplementary file 1 — Supplementary Information [file 41467_2020_20687_MOESM1_ESM.pdf]

Mathematical model of COVID-19 intervention scenarios for São Paulo- Brazil

In this Supplementary Information, we justify our assumption that São Paulo is a single, homogeneous mixing system, discuss in greater detail potential limitations of such assumption, and thoroughly describe our optimization workflow.

Supplementary Discussion

Homogeneously Mixing System Assumption

To verify the homogeneously mixing system assumption, we investigated how the COVID-19 daily cases and daily deaths in the state as a whole[1] correlated to the data of the city of São Paulo[2] (R^2=0.99, p<0.001 for all correlations; Supplementary Figure 1a,b show the time series).

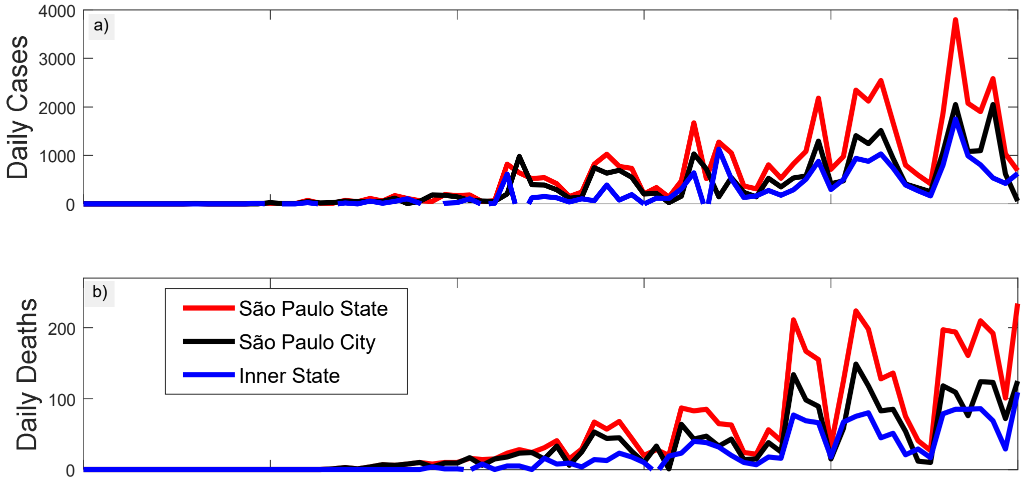

Supplementary Figure 1: **COVID-19 daily cases and daily deaths for the state of São Paulo, the city of São Paulo, and the inner state of São Paulo**. **a** Daily cases showing high correlation between São Paulo State and São Paulo City (R^2=0.91, $F_{1,84}$=849.014, p<0.001, 95% CI [1.458, 1.672]), São Paulo State and and Inner State (R^2=0.84, $F_{1,84}$=439.964, p<0.001, 95% CI [1.816, 2.196]) and São Paulo City and and Inner State (R^2=0.568, $F_{1,84}$=110.664, p<0.001, 95% CI [0.816, 1.196]). **b** Daily deaths showing high correlation between São Paulo State and São Paulo City (R^2=0.976, $F_{1,84}$=3438.668, p<0.001, 95% CI [1.593, 1.705]), São Paulo State and Inner State (R^2=0.951, $F_{1,84}$=1633.702, p<0.001, 95% CI [2.216, 2.446]) and São Paulo City and and Inner State (R^2=0.864, $F_{1,84}$=532.637, p<0.001, 95% CI [1.216, 1.446]).

However, part of the correlation found may be explained by reporting biases (e.g. weekend bias); thus, we further verified that there is limited spatial variation in the

pandemic progression in the state by comparing the COVID-19 cases' density graph on April 30, May 15, and May 30, 2020 (Supplementary Figure 2)[3].

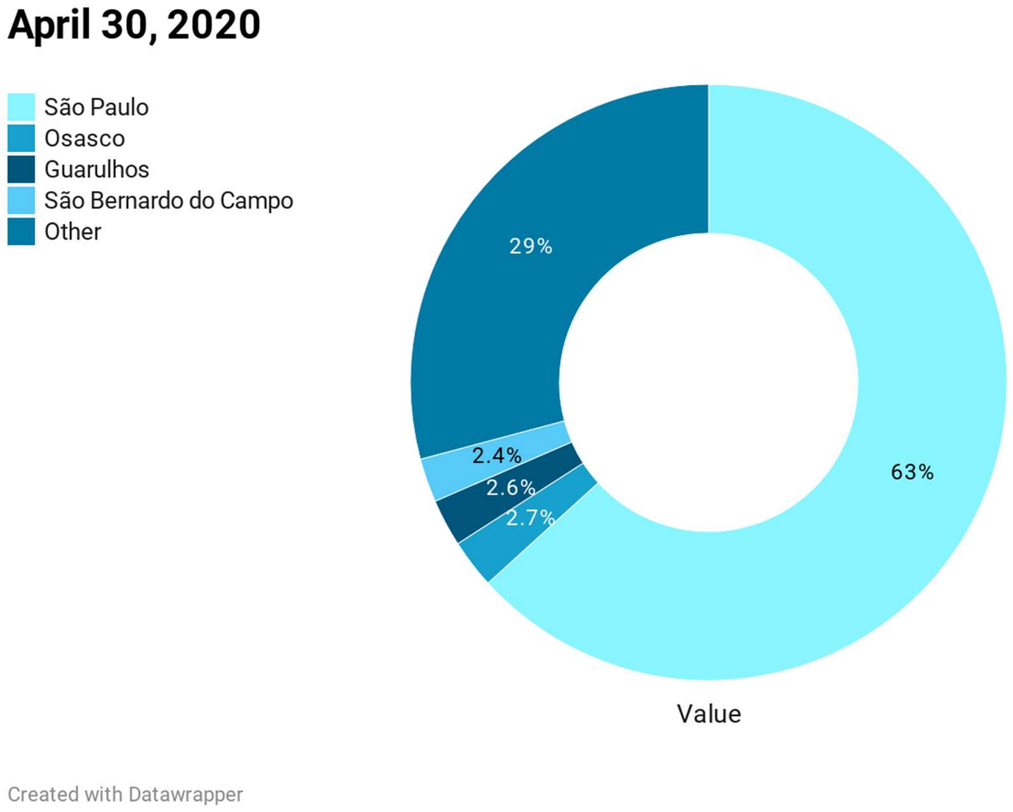

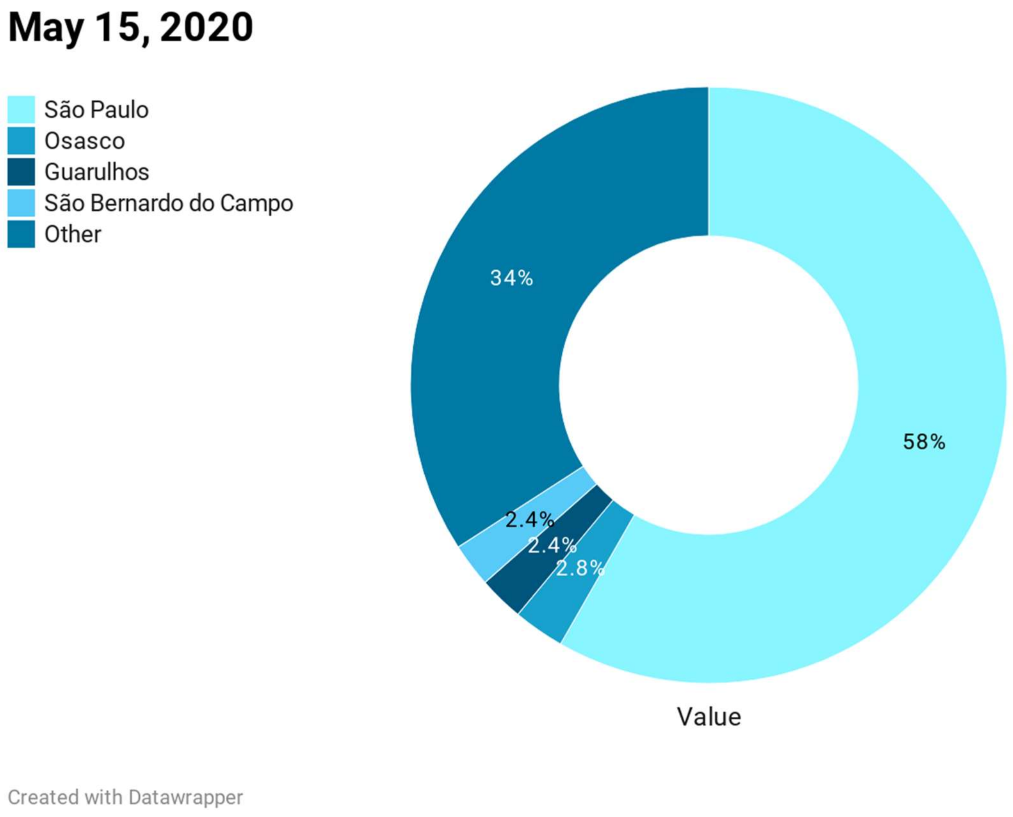

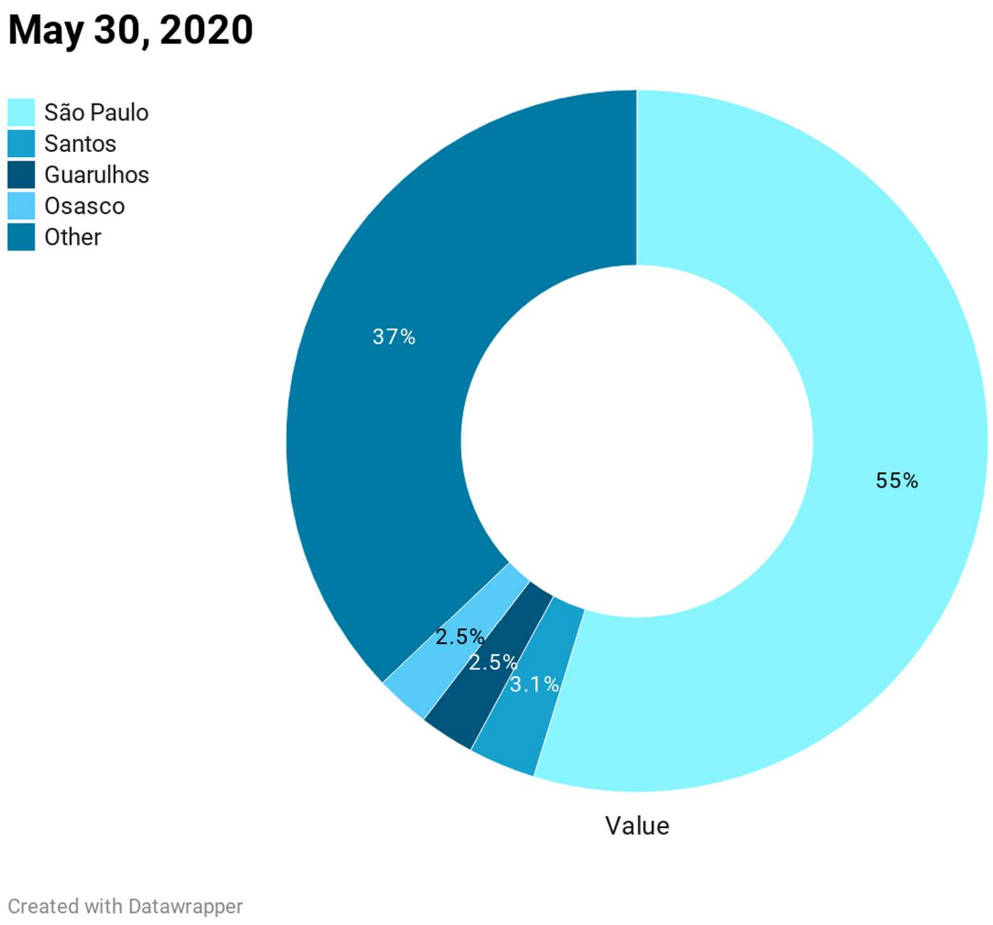

Supplementary Figure 2: COVID-19 state of São Paulo cases' multiple donuts chart on April 30, May 15, and May 30, 2020.

Finally, we verified the state's homogeneity in terms of population density[4], and that the state was isolated from other countries and Brazilian states during this pandemic period. With over 46 million residents, São Paulo is the most populous state in Brazil. Also, São Paulo has a large territorial dimension (248.209 km²), being larger than several countries worldwide, such as Portugal and The United Kingdom. Despite its size, however, the population distribution of São Paulo is quite homogeneous with a demographic density of 166.23 inhabitants/km², whereas the demographic density of Spain is 90 inhabitants/km² and France is 113.5 habitants/km², for example. Given the population density of São Paulo, it is not entirely surprising that it a pandemic hotspot with alarming rates of infections and associated deaths.

As for the isolation of the state, on March 20, 2020, the government decreed through the official gazette (Nº 64.879), a state of public emergency in São Paulo[5]. All non-essential activities were suspended until April 30. The order was later extended through June 15 (Nº 64.994)[6]. Besides, travel restrictions reduced air and land flow in and out of the state of São Paulo. São Paulo airports limited their flights or were closed for departures and arrivals (*e.g.* Congonhas) to mitigate the spread of COVID-19. From April 1-April 20 Congonhas had 126 flight arrivals and departures. In the same period last year, there were 9,489 flights. This difference represents a 98.68% drop in flights during the pandemic period[7]. With the data from the Operational Report of the Guarulhos Airport in São Paulo[8], which is the largest airport in Brazil, we made a graph comparing the movement (landings and takeoffs) between 2019 and 2020, demonstrating the drastic reduction in the number of flights as a result of the coronavirus pandemic.

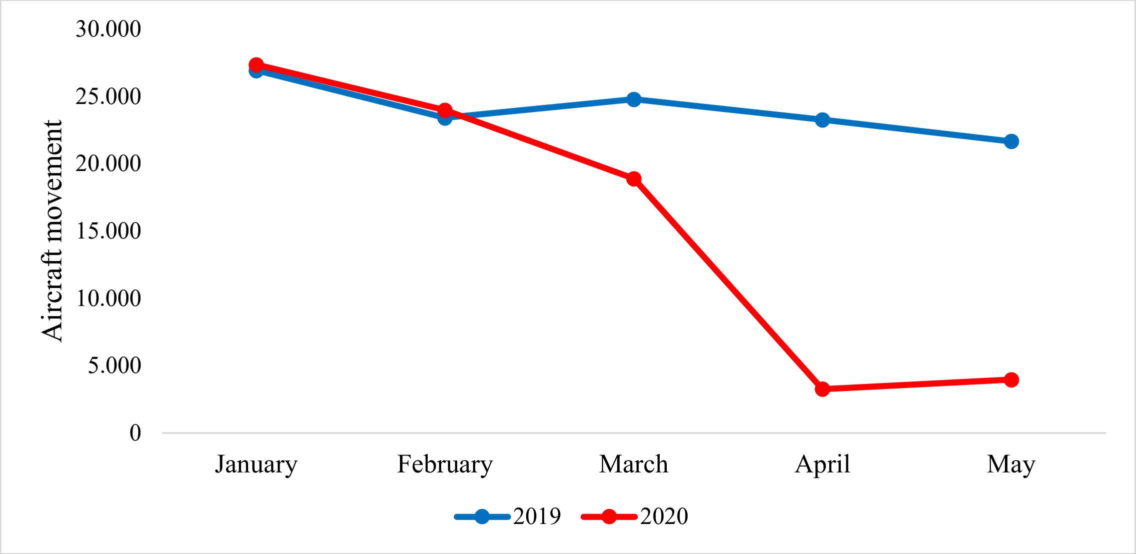

Supplementary Figure 3: Aircraft movement in 2019 and 2020 (Guarulhos Airport).

Also, the flow of trips at bus terminals had similarly reduced. Using information from the Observatory for Tourism and Events in São Paulo[9], we created a graph illustrating the reduction in the number of arrivals at the 3 main bus terminals in São

Paulo (Tietê, Barra Funda and Jabaquara) in March, April, and May, comparing 2020 with 2019.

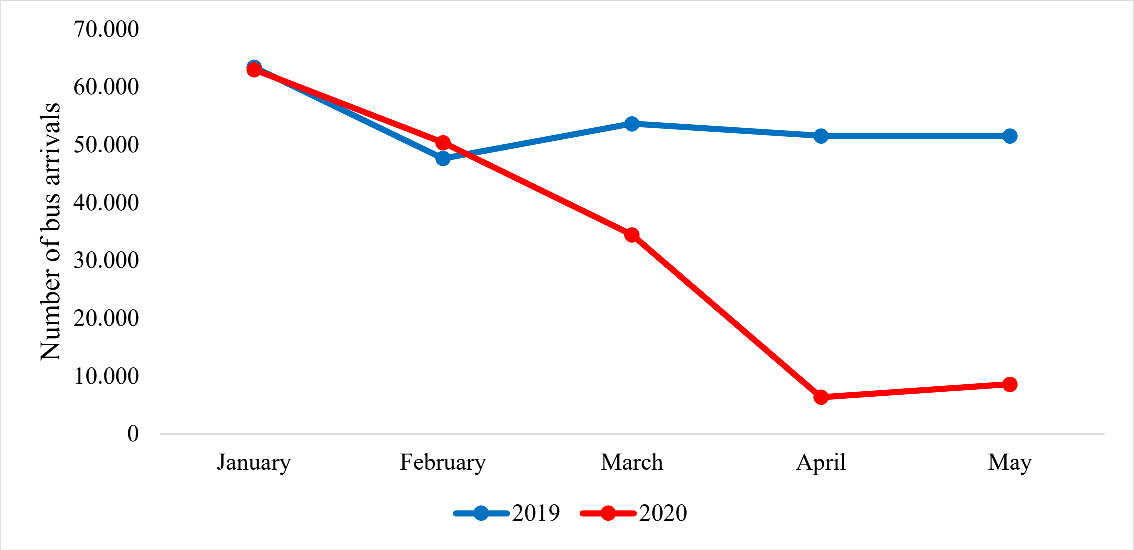

Figure 4: Numbers of bus arrivals in 2019 and 2020 in São Paulo.

Reduced travel in and out of São Paulo confirms São Paulo was relatively isolated from other countries and states within Brazil during the pandemic period.

Limitations of a Homogeneously Mixing System Assumption

Although we have shown strong evidence that there is limited spatial variation in the pandemic progression in the state and that the state was isolated from other countries and Brazilian states during this pandemic period, it may be argued that no system is 100% homogenous nor isolated. Furthermore, considering there was a great decrease in mobility, especially driving and transit, across all the state, it is possible that the homogeneity assumption may be partially violated at a city or neighborhood level, and that, at some parts of the state, the pandemic spread could be contained within localized pockets.

To verify the impact of possible localized pockets within the state on the disease's progression estimated by our model, we ran a sensitivity analysis. First, we considered having all daily cases and daily deaths recorded within a certain percentage of the

population (25, 50, 75%) isolated completely from the rest. Second, we considered a more localized spread could result in localized increases in acquired immunity that may influence social distancing. Thus, we simulated each scenario considering 50, 75, and 100% of the magnitude of the SD time series used in the study till the present day (Supplementary Figure 5). To clarify, for example, 50% of the magnitude of the SD time series implies using a value of SD of 30% in a day where our mobility estimations from google and apple were 60%. Throughout all scenarios, α was kept constant, Npop and SD were manipulated, and all other model parameters were optimized, for each scenario, as described in the main document's "Model's Coefficients Optimization." Future values of social distancing and protection, for simplicity, were chosen to stay at current levels. Supplementary Figure 5b,c indicates that, at 50 and 75% SD magnitude, having all cases concentrated in 75% of the state yielded similar results to having the whole SD magnitude and state population entered to the model; in other words, our model future progressions reported are reliable if disease-free isolated parts of the state do not exceed approximately 25% of the state's population. All these conclusions are drawn considering a different set of parameters for each scenario after individual fitting of the model to real data. If the same set of parameters chosen in our main results section were kept and only SD and Npop were manipulated, the conclusions would be different. In this case, both reducing SD and Npop would cause clear proportional changes in the number of infections and critical cases (Equation 1 from the main document).

Additionally, the sensitivity analysis indicates that, at 50 and 75% SD magnitude, if disease-free isolated parts of the state include more than 25% of the population (e.g. Supplementary Figure 5: 25% Npop - blue line and 50% Npop - green line) our results and projections may be over-estimations. Finally, our results may have been under-estimations if there are localized pockets within the state, but they do not influence the

impact of social distancing (Supplementary Figure 5a). In such a case, the worst-case scenario, or the case with the highest estimated peak of critical cases, happened with 100% SD and *50% of* Npop.

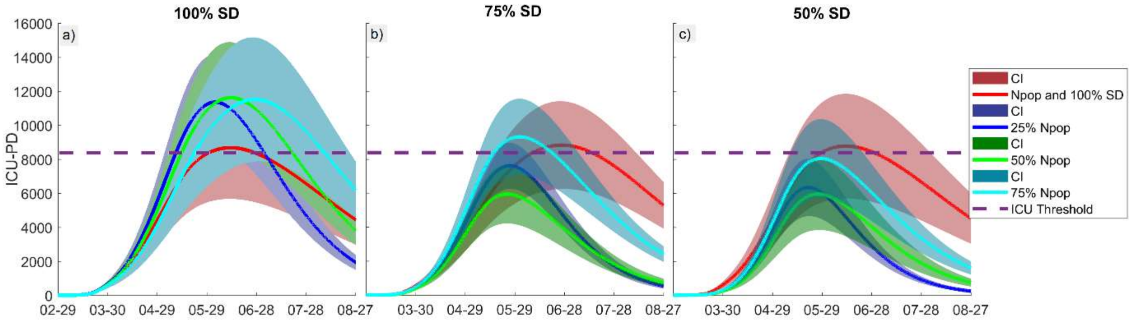

Supplementary Figure 5: Sensitivity analysis considering having all daily cases and daily deaths recorded within a certain percentage of the population (25, 50, 75%) isolated completely from the rest. Each scenario was simulated considering 100% (a), 75% (b), and 50% (c) of the magnitude of the social distancing (SD) with protection rate (α) kept constant across scenarios and all other model parameters optimized for each scenario. Future values of social distancing and protection were chosen to stay at current levels. For comparison, it also shows no part of the population was isolated (Npop and 100% SD). Npop stands for the state population.

To test the reliability of our future projections' conclusions in terms of protection and mitigation SD strategy, we considered the worst-case scenario to run the optimization workflow, as described in the "Future Projections" subsection. Again, we minimized ICU_E, ICUE_1, ICUE_2, and SD to find the optimal mitigation strategy and protection ranges that would yield control over the pandemic. The results (Supplementary Figure 6) indicate the same conclusions as those described in the results section of the main document. Based on this result, we believe that, in the worst-case scenario of Supplementary Figure 5, our model future progressions' conclusions in terms of strategy are reliable.

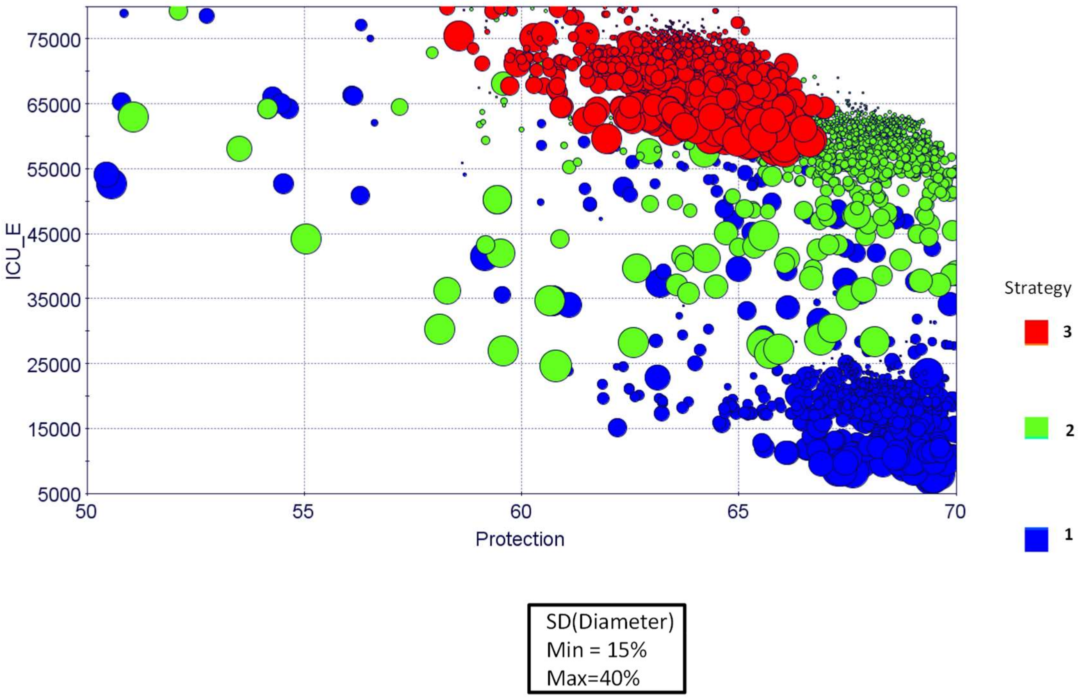

Supplementary Figure 6: Optimization results for the worst-case scenario of the sensitivity analysis of Supplementary Figure 5, considering having all daily cases and daily deaths recorded within 50% of the population and 100% of the magnitude of social distancing (SD). It shows the influence of mitigation Strategy (color) and Protection (% x-axis) on the total number of critical cases over the ICU threshold (ICU_E - y-axis) for the whole period of analysis (end day Dec 25, 2021). Red color indicates constant SD strategy, green color indicates intermittent strategy, and blue color indicates a stepping down strategy. SD values are shown from 15-40% by the diameters of the circles.

Supplementary Methods

The Optimization Framework

In this part of the Supplementary Information, we detail the optimization workflow using ESTECO’s mode Frontier (Esteco s.p.a; 2017R4-5.6.0.1). Optimization was implemented to find the optimal mitigation strategy.

An optimization workflow was implemented to study the model further and to optimize the parameters. ESTECO's modeFrontier was used to wrap the SEIR model and

automate the analysis. The workflow implemented is shown in Supplementary Figure 7, along with a brief explanation of the workflow regions.

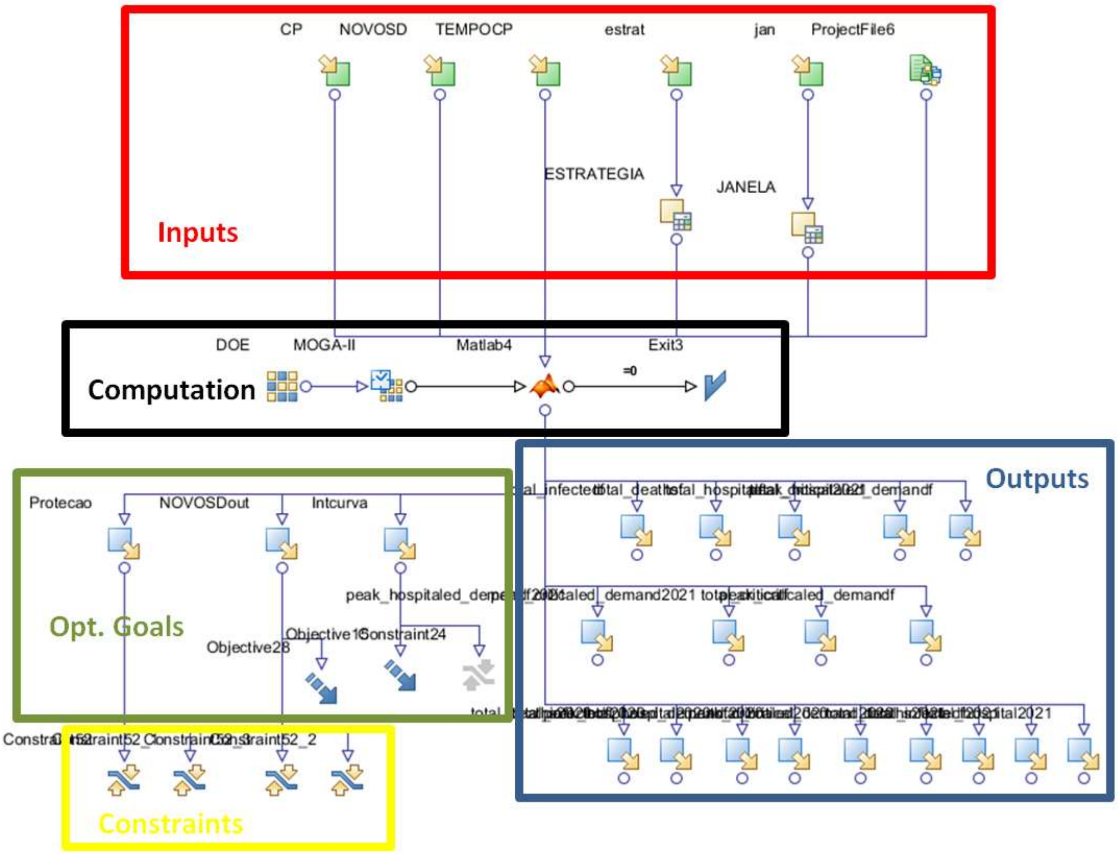

Supplementary Figure 7: The implemented workflow:

- Inputs: Variables that will be changed during the optimization study.
- Computation: Process control, DOE definition, and optimization algorithm selection.
- Optimization Goals: Output variables of the model that will be optimized.
- Constraints: Constraints applied to the output variables during the optimization solution.
- Outputs: Other observable states and variables of the models useful for post-processing.

The strategy for studying and optimizing the model consisted of running a DOE (Design of Experiment) scheme of approximately 2000 total individuals with a mix of SOBOL, Latin Hypercube, and Latin Square individuals. This scheme was chosen to better represent the design space without the need for a full-factorial solution. A full-factorial solution would be computationally expensive.

The DOE (Design of Experiments)

As best practice on the Design of Experiments, the least possible correlation between input variables was sought out and guaranteed to achieve values lower than 0.02.

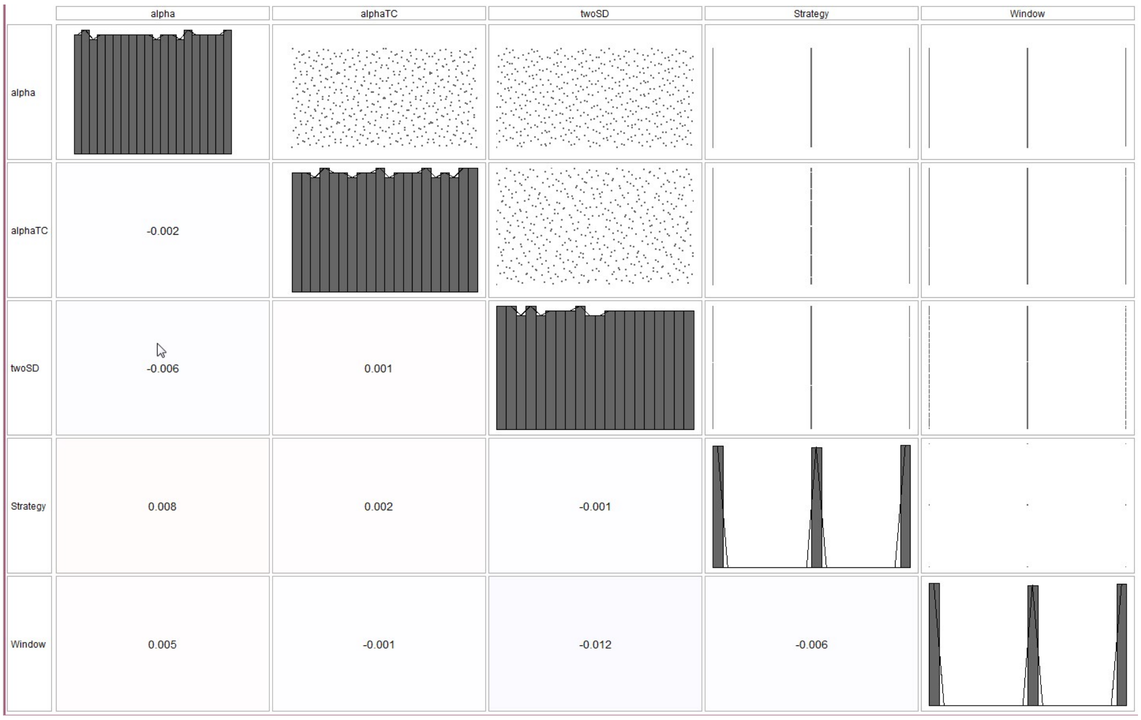

Supplementary Figure 8: Design of Experiments with corresponding correlation values.

DOE Results - SP

Following, we show the results of the correlation scatter matrix of the DOE for São Paulo as an example (Supplementary Figure 9).

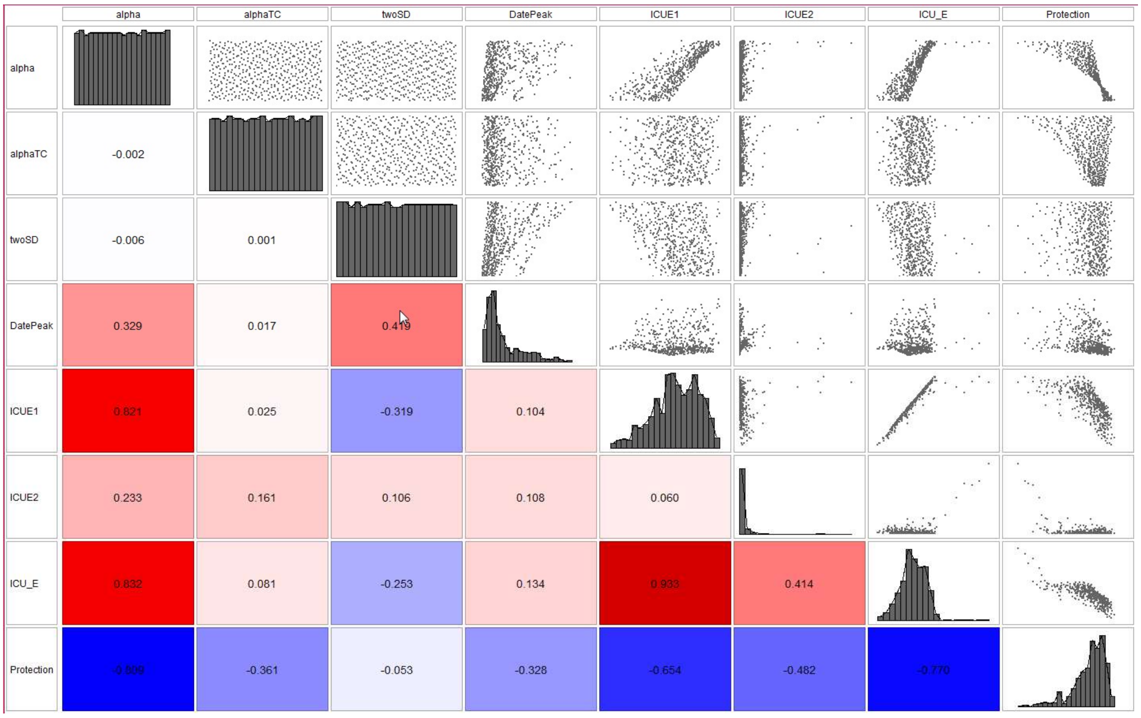

Supplementary Figure 9: Results of the correlation scatter matrix of the DOE for São Paulo. The darker the color, the strongest the mathematical correlation. RED indicates a positive correlation; BLUE indicates a negative correlation. The diagonal shows the histograms of each variable. The matrix's upper triangular region shows the scatter plot of the Column variable (X-axis) versus the Line Variable (Y-axis).

Even though a numerical correlation was found to be high, the scatter plot suggests that second and third-order effects affect the output of ICU_E.

Optimization - SP

After analyzing the DOE, the next step was to run a multi-objective optimization of the model, seeking to Minimize the variable ICU_E, ICUE_1, and ICUE_2 and Social Distancing. These two variables are in direct opposition, and this strategy should yield a Pareto frontier of optimum solutions. One studying the public health strategy might then use this Pareto frontier to choose an optimum solution for his/her local reality.

As a first approach to the optimization problem, the solution was unconstrained so the algorithm could select freely within the search space.

A MOGA - Multi-Objective Genetic Algorithm was used to drive the optimization due to the discrete nature of the variables Strategy and Window. It would be rather

difficult for a gradient-based algorithm to find a gradient solution with such types of variables.

Optimization Results - Global SP (unconstrained)

The result of the unconstrained optimization, i.e. with the same boundaries as the DOE, is shown here:

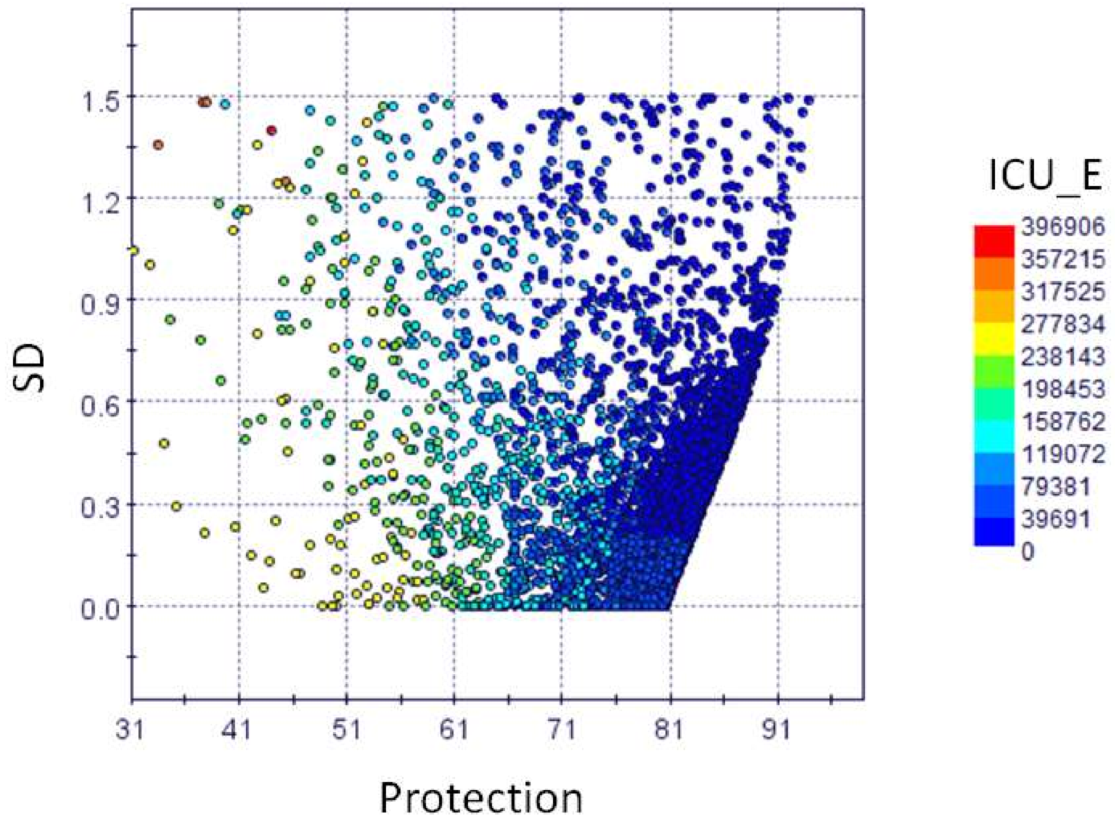

Supplementary Figure 10: Result of unconstrained optimization.

Two opposing design variables, driving the so-called Pareto frontier. The area density of points in the plot shows how the MOGA Algorithm uses evolution pressure to drive the individuals towards the Pareto frontier.

This plot also shows a secondary Pareto front (marked green). Since the Strategy and Window are combinatorial discrete solutions, it will be shown later that this is the case: for every combination of Strategy and Window, there is a well-defined Pareto frontier.

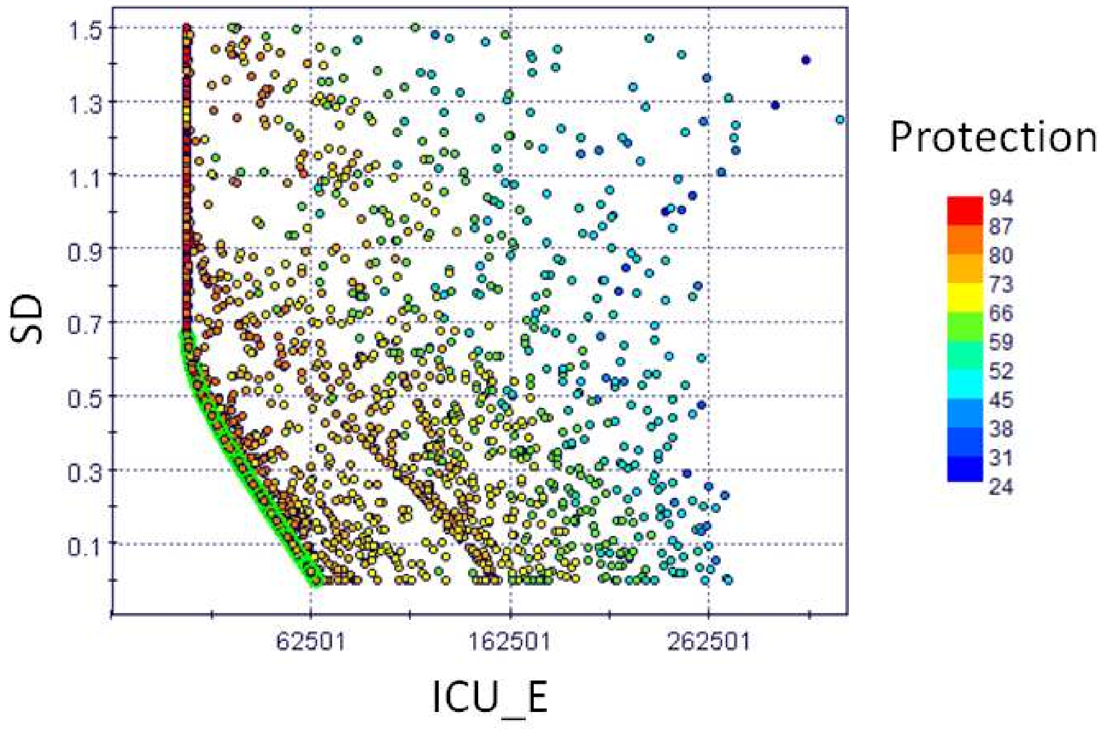

Supplementary Figure 11: Result of the unconstrained optimization showing two opposing design variables, driving a Pareto frontier, marked in green.

These SD and Protection levels might be considered unrealistic for both the SP and Brazilian realities, so a Constrained optimization was then used to find an optimal solution, which is also feasible and realistic. In such a scenario, the Pareto front can and has found solutions that can be misleading in Window and Strategy.

Results - Constrained SP

SD and Protection Levels have been constrained to 30 to 80% and 50 to 70%, respectively, which are arguably reasonable values for the Brazilian and SP reality.

By constraining the optimization problem, a change in best solution (Strategy and Window) was observed since the search space was confined within the constraints. From the MOGA algorithm standpoint, results that violate the constraints are not chosen to yield offspring to the next generation, thus reducing the likelihood of a certain gene to be perpetuated. This evolutionary pressure drives the results within the constrained boundaries, creating new Pareto frontiers that are feasible and best suited for the

constrained reality. Supplementary Figure 12 shows all results, both the constrained and the unconstrained.

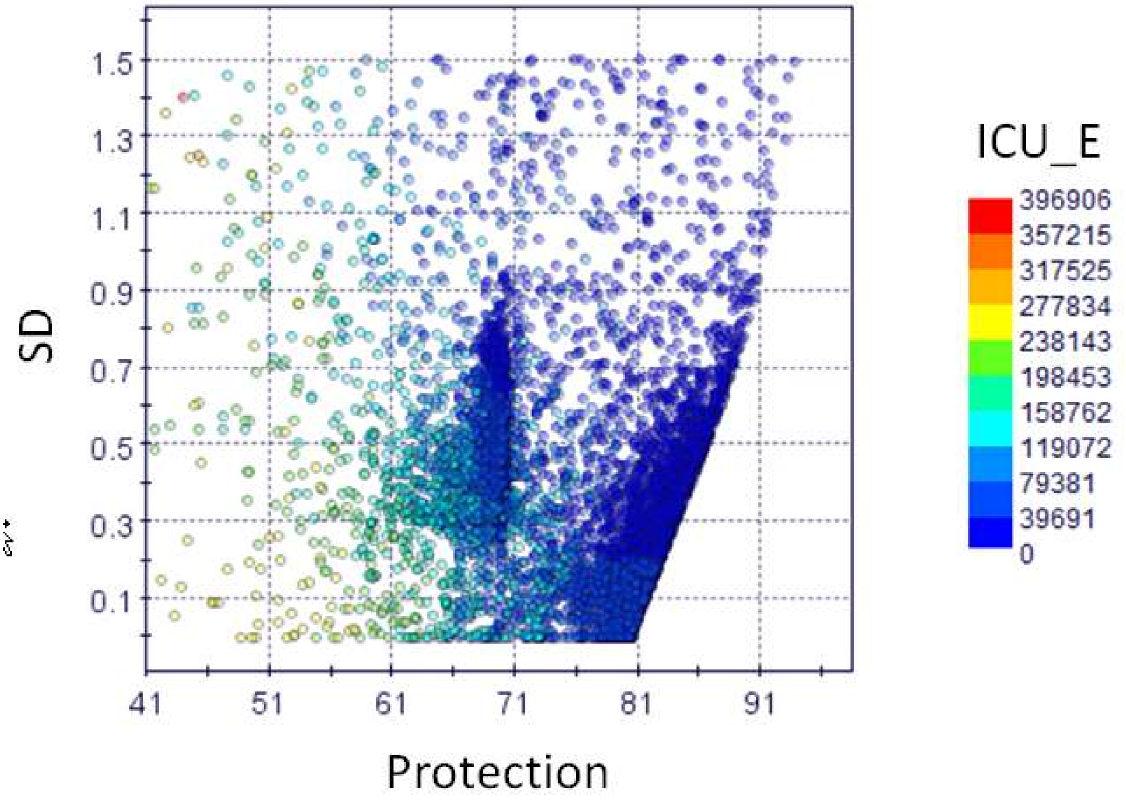

Supplementary Figure 12: Constrained and unconstrained results.

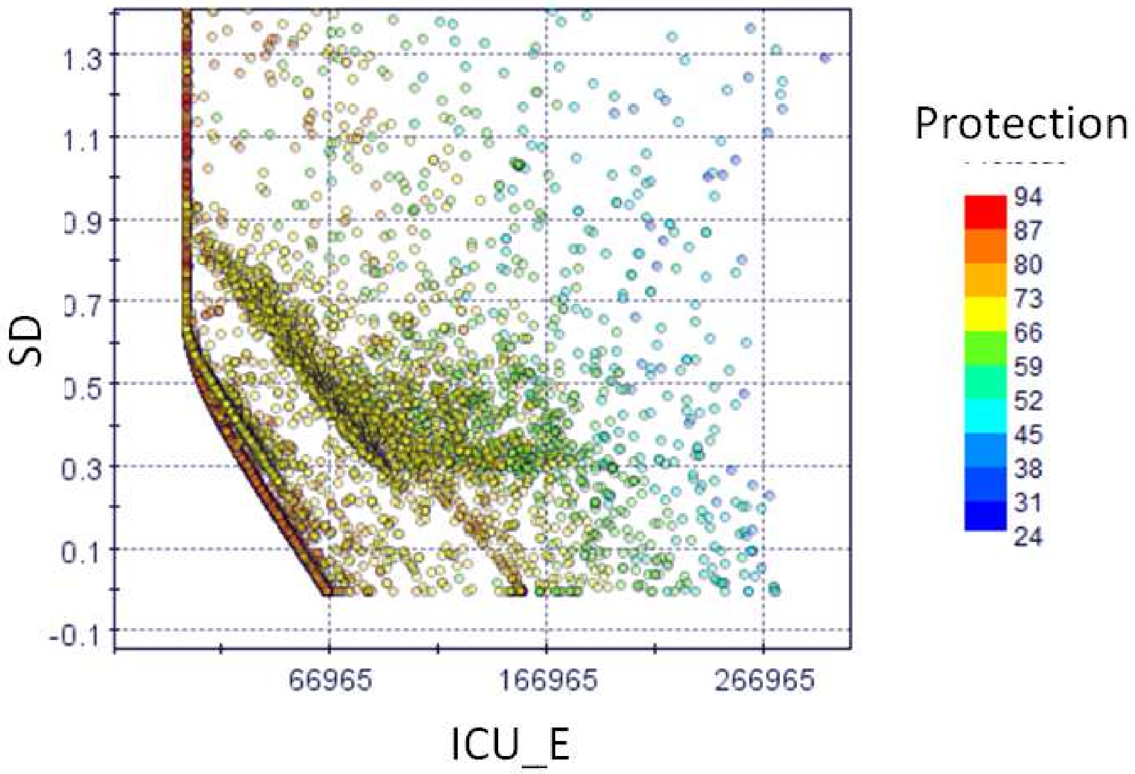

Supplementary Figure 13: Constrained and unconstrained results with Pareto frontier.

Supplementary Figure 14 shows only the results that fall within the constraints.

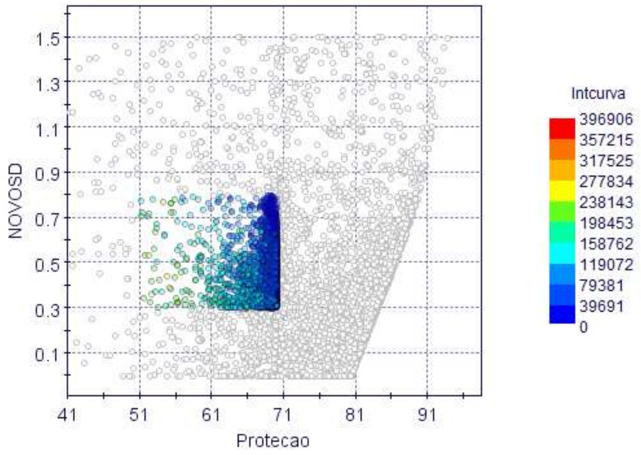

Supplementary Figure 14: Constrained results.

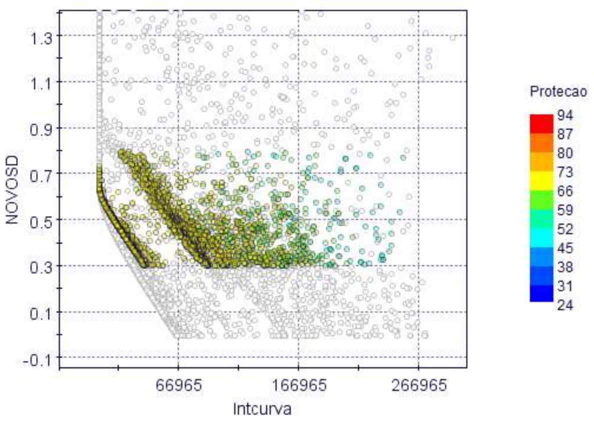

Supplementary Figure 15: Constrained results and Pareto frontier.

Not only the Pareto frontier has been changed, but now two very distinct frontiers are visible. This could have been caused either by a combination of Strategy and Window or by a local minimum during the optimization overcome by the Genetic Algorithm.

To further understand this phenomenon, a discussion on the influence of the Strategy and Window variables is proposed, and the results are presented in the main study.

MOGA and Convergence Efficiency

For the convergence of the optimization strategy, ICU_E was chosen as the most important variable to be monitored, as can be seen:

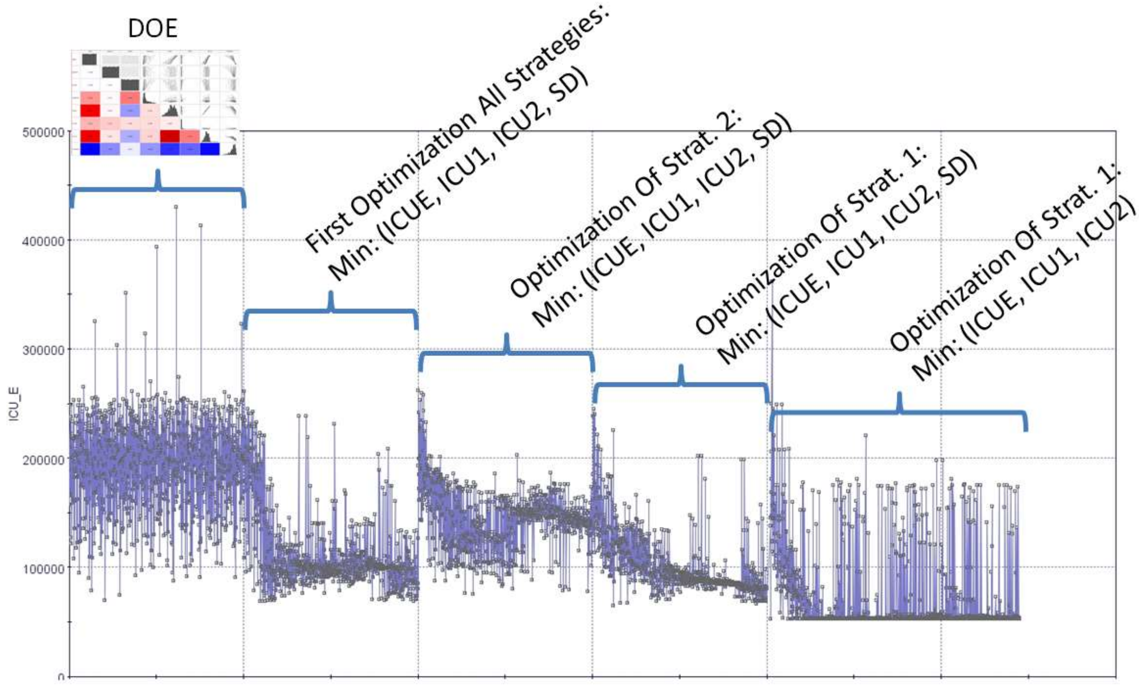

Supplementary Figure 16: convergence of the optimization strategy

It can be observed as each step shows the convergence of the GA towards the desired solution, and therefore, the results shown in the main study are considered in the state are robust from the optimization standpoint.

Supplementary References

1. Fundação Sistema Estadual de Análise de Dados Estatísticos (SEADE). (2020); https://www.seade.gov.br/coronavirus/

2. Prefeitura de São Paulo – Vigilância em Saúde. (2020); https://www.prefeitura.sp.gov.br/cidade/secretarias/saude/vigilancia_em_saude/index.php?p=295572

3. Fundação Sistema Estadual de Análise de Dados Estatísticos (SEADE) - Repository. (2020); https://github.com/seade-R/dados-covid-sp

4. Instituto Brasileiro de Geografia e Estatística. (2010); https://www.ibge.gov.br

5. Assembleia Legislativa do Estado de São Paulo. (2020); https://www.al.sp.gov.br/norma/193347

6. Assembleia Legislativa do Estado de São Paulo. (2020); https://www.al.sp.gov.br/repositorio/legislacao/decreto/2020/decreto-64994-28.05.2020.html

7. Portal Globo de Notícias. (2020); https://g1.globo.com/sp/sao-paulo/noticia/2020/04/27/aeroportos-de-congonhas-e-cumbica-tem-patios-com-avioes-parados-e-queda-no-numero-de-voos-durante-pandemia-em-sp.ghtml

8. Relatório Operacional do Aeroporto de Guarulhos. (2020); https://www.gru.com.br/pt/RelatorioOperacional/2019-05.pdf

9. Observatório do turismo de São Paulo. (2020); http://www.observatoriodoturismo.com.br/pdf/rodoviarias_maio_2020.pdf
